# Retinal Vein Changes as a Biomarker to Guide Diagnosis and Management of Elevated Intracranial Pressure

**DOI:** 10.3389/fneur.2021.751370

**Published:** 2021-10-18

**Authors:** Heather E. Moss

**Affiliations:** ^1^Department of Ophthalmology, Stanford University, Palo Alto, CA, United States; ^2^Department of Neurology and Neurological Sciences, Stanford University, Palo Alto, CA, United States

**Keywords:** intracranial pressure, retinal vein, idiopathic intracranial hypertension (IIH), spontaneous retinal venous pulsation, ophthalmodynamometer, biomarker

## Abstract

Retinal vein changes, which can be observed on clinical exam or ophthalmic imaging, are promising non-invasive biomarkers for elevated intracranial pressure (ICP) as a complement to other markers of high ICP including optic nerve head swelling. Animal and human studies have demonstrated increase in retinal vein pressure associated with elevated ICP mediated by increase in cerebral venous pressure, compression of venous outflow by elevated cerebral spinal fluid pressure in the optic nerve sheath, and compression of venous outflow by optic nerve head swelling. Retinal vein pressure can be estimated using ophthalmodynamometry. Correlates of retinal vein pressure include spontaneous retinal venous pulsations, retinal vein diameter, and retinal vein tortuosity. All of these have potential for clinical use to diagnose and monitor elevated ICP. Challenges include diagnostic prediction based on single clinical measurements and accurate assessment of retinal vein parameters in cases where optic nerve head swelling limits visualization of the retinal veins.

## Introduction

Elevated intracranial pressure (ICP) is a pathophysiological state, detection of which is relevant to the diagnosis of neurological and neurosurgical diseases such as brain tumors, cerebral venous sinus thrombosis, and idiopathic intracranial hypertension (IIH). Monitoring of ICP is relevant to mitigating the risk of vision loss due to optic nerve dysfunction caused by elevated ICP. However, current methods for measurement of ICP are limited to invasive techniques. Ophthalmic changes are promising non-invasive biomarkers of elevated ICP since they can be seen on clinical exam and ophthalmic imaging.

Papilledema, swelling of the optic nerve head due to axoplasmic stasis in the retinal ganglion cells that form the optic nerve, is a downstream effect of high ICP (1). Accordingly, development and resolution of papilledema as measured on clinical exam and ophthalmic imaging is a surrogate marker for ICP changes that is used clinically. However, papilledema changes are delayed following ICP changes, likely due to biological dynamics of axoplasmic stasis and delayed equilibration between optic nerve sheath intracranial subarachnoid space compartments (2, 3). This leads to uncertainty when evaluating ICP treatment response on the basis of optic nerve appearance. Thus, there is a need for additional biomarkers that better capture ICP dynamics to complement observations of the optic nerve and guide clinical care.

Retinal vein changes are promising biomarkers of elevated ICP because, like optic nerve changes, they are observable on clinical exam and ophthalmic imaging but, unlike optic nerve appearance, change rapidly following ICP changes. There are early data to support the potential of retinal vein changes as a biomarker to aid in diagnosing and monitoring high ICP states.

## Mechanism of Retinal Vein Pressure Increase in Elevated ICP States

To consider retinal vein measurements as potential markers of elevated ICP, it is necessary to understand the broader landscape of parameters that influence retinal vein appearance. As with all blood vessels, retinal veins are compliant tubes. Their appearance is based on anatomical branching pattern, compliance of the vessel wall, pressure on the outside [i.e., intraocular pressure (IOP)], and pressure of their contents (i.e., blood). Blood pressure and flow are determined by upstream pressure, downstream pressure, and resistance. For retinal veins, the relevant pressures are central retinal artery pressure and central retinal venous pressures with resistance being mainly in the retinal arterioles. However, these pressures do not exist in isolation and are impacted by upstream and downstream vascular pressures (e.g., cerebral and systemic arterial and venous pressures) and resistances.

Elevated ICP impacts retinal veins through three mechanisms. First, it is associated with changes in downstream cerebral venous pressures. Second, elevated pressure in the cerebral spinal fluid (CSF) in the subarachnoid space inside the optic nerve sheath exerts external pressure on the central retinal vein as it exits the eye, which increases vascular resistance. Finally, elevated pressure in the CSF in the subarachnoid space compresses the retinal ganglion cells in the optic nerve, and the feeding arteriolar supply causes papilledema to develop. Papilledema also exerts external pressure on the central retinal vein which increases vascular resistance.

Non-human primate studies of acute ICP elevation have confirmed the first two mechanisms with the following observations. Superior ophthalmic vein pressures are elevated in association with ICP elevation (4). Increases in retinal vein pressure associated with ICP increases are blunted by ligating the optic nerve sheath to limit transmission of elevated ICP to the optic nerve sheath (5). It is notable that papilledema did not develop in these experiments, reinforcing that high ICP can induce retinal vein changes in the absence of papilledema. No similar observations have been reported in humans, perhaps due to lack of an experimental model of acute ICP elevation.

Observation of pathological retinal findings in humans with elevated ICP support an association between high ICP and elevated retinal venous pressures in humans. Examples include venous stasis retinopathy (6) and retinal-choroidal venous collaterals (7). However, these occur in a minority of individuals and therefore are not candidates as biomarkers.

## Ophthalmodynamometry

**Figure 1:**
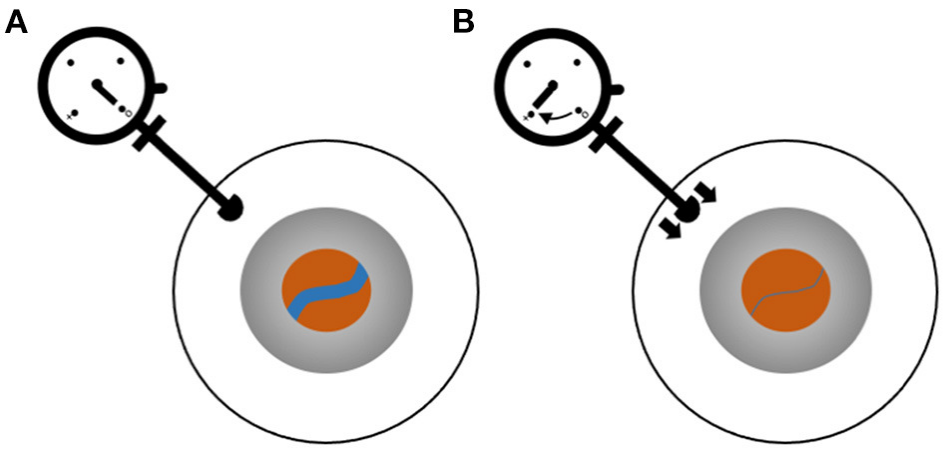
Ophthalmodynamometry for measurement of retinal venous pressure. Ophthalmodynamometer device consists of a probe with which pressure can be applied to the sclera and a dial indicating the pressure. The eye is shown schematically as three circles representing the sclera (white), iris (gray), and retina visualized through the pupil (orange). The blue line is a retinal vein. **(A)** Baseline, no force being applied to the eye. **(B)** Pressure is applied by pushing the ophthalmometer against the sclera, and the vein diameter is observed to decrease. The pressure at which the vein collapses is recorded.

First developed in the early twentieth century, ophthalmodynamometry involves application of force to the external eye and observing the retinal vessels as force is increased. The force at which a vessel collapses is indicative of pressure in the upstream vessel (8). The components needed to perform this procedure are a device that can measure applied force to the globe and a method to visualize the retinal vessels (Figure 1). Historically, the device used to apply and measure force was mechanical, and direct or indirect ophthalmoscopy was used to visualize the vessels (9). Though digital methods of measuring force (10) have since been implemented, the technique is mainly used in the research setting.

Early use of ophthalmodynamometry focused on arterial pressure measurements. More recently, multiple studies of ophthalmodynamometric measurements in patients undergoing invasive ICP monitoring have shown ICP and retinal vein pressure to be highly correlated (11, 12). However, sampling of the ICP range was uneven, and these correlations were driven by a few subjects with high ICP. The false negative rate was higher than desirable with 6 of 22 subjects with high ICP not having elevated retinal vein pressures by ophthalmodynamometry in the larger study (11). Reinforcing the important role that papilledema plays in increasing retinal vein outflow resistance, subjects with papilledema were noted to have elevated retinal vein pressures even after ICP was surgically lowered (11). This persisted until papilledema resolved.

## Spontaneous Retinal Venous Pulsations

Spontaneous retinal venous pulsations, visualized as an oscillation in retinal vein diameter, often near the optic disc margin, are a result of IOP and ICP oscillations leading to transmural pressure oscillations causing partial collapse of the vein (13). When retinal vein pressure is elevated, the amplitude of transmural pressure oscillations is reduced, and visible pulsations do not manifest. This is the explanation given for the clinical observation of absence of spontaneous retinal venous pulsations in association with high ICP states. Animal models have experimentally confirmed the association between acute ICP elevation and loss of spontaneous retinal venous pulsations (14).

The detection of spontaneous retinal venous pulsations is often used as a clinical screening tool to rule out elevated ICP, although it is an imperfect one with the presence of spontaneous retinal venous pulsations assessed by an expert examiner having sensitivity approaching 90% to exclude high ICP but specificity of <20% to detect high ICP (15). Some of this is due to moderate inter-observer agreement. However, a study using video infra-red fundus imaging with expert review to detect spontaneous retinal venous pulsations in subjects undergoing invasive ICP monitoring reported a similar receiver operating characteristic area under the curve of 0.8 (16). Thus, our model of a direct relationship between spontaneous retinal venous pulsations and ICP is likely oversimplified. Non-human primate models have potential to address this knowledge gap (17).

## Retinal Vein Diameter

The diameter of a compliant tube increases as transmural pressure is increased (18), and accordingly, retinal vein diameter is a potential marker for retinal vein pressure and, by extension, ICP. Compared to ophthalmodynamometric measurements or spontaneous retinal venous pulsation detection, retinal vein diameter measurement is complicated by the need for image calibration. This is typically addressed by using axial length, camera, and refractive error to calibrate the images. An alternative is relative scaling, for example, a ratio compared to artery diameter or optic disc diameter. However, this latter scale is not useful when papilledema is present since this obscures the optic nerve border.

Qualitative (5) and quantitative (14) retinal vein diameter increases have been reported in association with acute ICP increases in animal models without papilledema. Association between elevated ICP and retinal vein diameter in humans has been demonstrated in a variety of settings. In humans with IIH and papilledema, retinal vein diameters were larger than in subjects with pseudo-papilledema or normal optic nerves (19). Retinal vein diameter decrease has been demonstrated within 1 h following CSF drainage (20), 1 month after optic nerve sheath fenestration (21), and following medical or surgical treatment with improvement in symptoms (19).

The largest study of retinal vein diameter in IIH was performed in conjunction with the IIH Treatment Trial (IIHTT), a randomized controlled trial of acetazolamide + weight loss intervention vs. placebo + weight loss intervention in 165 subjects with IIH, papilledema, and mild vision loss (22). At baseline, retinal arterial:venous diameter ratio was inversely associated with measures of papilledema on fundus photography (23). In other words, vein diameter increased as optic nerve swelling increased. After 6 months of treatment, there was a decrease in retinal vein diameters overall. This decrease was associated with baseline optic nerve head volume, baseline ICP, and change in optic nerve head volume in bivariate models (24). In multiple variable models only change in papilledema was associated with retinal vein diameter decrease.

Thus, while there is clear evidence that ICP in the absence of papilledema can impact retinal vein diameter, likely mediated *via* downstream venous pressure increases and optic nerve sheath pressure increases, the additional mechanism of papilledema causing downstream venous resistance increases may be a stronger factor. However, this does not negate the potential of retinal vein diameter as a marker of ICP elevation. Rapid retinal vein changes following CSF pressure lowering (20) suggest retinal vein-based biomarkers may complement papilledema-based biomarkers by detecting early changes.

The IIHTT data set is unique from the others used to study spontaneous retinal venous pulsations or ophthalmodynamometry as retinal vein-based markers of elevated ICP in that all subjects had papilledema covering the full Frisen scale. Of the 126 subjects that completed follow-up, peripapillary vein diameter measurement in all four quadrants was possible in 92 (73%). This reflects retinal vasculature distortion by papilledema including obscuration of vessels and hemorrhages obscuring the branching pattern and illustrates a practical challenge in the use of peripapillary retinal vein measures as biomarkers of ICP.

## Retinal Vessel Tortuosity

In compliant tubes, increases in pressure initially cause an increase in diameter but subsequently cause an increase in tortuosity (18). Retinal vein diameter increase and tortuosity are typical findings in central retinal vein occlusion, resulting from increased retinal venous pressure due to downstream occlusion (25). However, while tortuosity is anecdotally observed in association with papilledema and is observed to improve in association with papilledema resolution following treatment, it has not been systematically studied.

## Discussion

There are multiple retinal vein findings that relate to ICP and show promise as clinical biomarkers of elevated ICP to complement those already used in clinical care. These include ophthalmodynamometry to measure retinal vein pressure, detection of spontaneous retinal venous pulsations, measurement of retinal vein diameter, and measurement of retinal vein tortuosity. Future research is needed to advance these to clinical care beyond a qualitative screening test as spontaneous retinal venous pulsation detection is currently used. Effort is needed to better understand the factors causing measurement variability and to develop measurement techniques less susceptible to failure due to papilledema.

There are multiple parameters impacting retinal vein pressure and direct/indirect measurements of this. While many of these have been identified, how they combine with each other to impact measurements has not been elucidated. Ophthalmodynamometric (11), non-human primate (5), and IIHTT retinal vein diameter (24) studies reinforce that ICP mediates retinal vein changes through multiple pathways, including cerebral hemodynamics impacting downstream venous pressure, optic nerve sheath subarachnoid pressure compressing the central retinal vein to increase venous outflow resistance, and papilledema compressing the central retinal vein to increase venous outflow resistance. Larger sample sizes in human studies including papilledema and animal studies that facilitate manipulation of independent variables are important to define the important relationships to move beyond the study of associations to clinical utility.

Distortion of the optic nerve and peripapillary retina by papilledema introduces a technical challenge when translating retinal vein measurement techniques developed for application in situations without optic nerve swelling. Potential solutions to improve feasibility of measurements include measurement of vessels distant from the optic nerve, for example by examining macular vessels or using widefield imaging.

## Author Contributions

HM contributed to conception and design of the study and wrote the manuscript.

## Funding

This study was supported by National Institutes of Health R21 EY 031726 and P30 EY 026877, Unrestricted Grant from Research to Prevent Blindness to the Stanford Department of Ophthalmology.

## Conflict of Interest

The author declares that the research was conducted in the absence of any commercial or financial relationships that could be construed as a potential conflict of interest. The handling editor declared a past collaboration with one of the author, HM.

## Publisher's Note

All claims expressed in this article are solely those of the authors and do not necessarily represent those of their affiliated organizations, or those of the publisher, the editors and the reviewers. Any product that may be evaluated in this article, or claim that may be made by its manufacturer, is not guaranteed or endorsed by the publisher.
